# A Comprehensive Investigation of Interactions between Antipsychotic Drug Quetiapine and Human Serum Albumin Using Multi-Spectroscopic, Biochemical, and Molecular Modeling Approaches

**DOI:** 10.3390/molecules27082589

**Published:** 2022-04-18

**Authors:** Seema Zargar, Tanveer A. Wani, Nawaf A. Alsaif, Arwa Ishaq A. Khayyat

**Affiliations:** 1 Department of Biochemistry, College of Science, King Saud University, P.O. Box 22452, Riyadh 11451, Saudi Arabia; szargar@ksu.edu.sa (S.Z.); aalkhyyat@ksu.edu.sa (A.I.A.K.); 2 Department of Pharmaceutical Chemistry, College of Pharmacy, King Saud University, P.O. Box 2457, Riyadh 11451, Saudi Arabia; nalsaif@ksu.edu.sa

**Keywords:** quetiapine, human serum albumin, hydrophobic interaction, thermodynamic parameters

## Abstract

Quetiapine (QTP) is a short-acting atypical antipsychotic drug that treats schizophrenia or manic episodes of bipolar disorder. Human serum albumin (HSA) is an essential transport protein that transports hormones and various other ligands to their intended site of action. The interactions of QTP with HSA and their binding mechanism in the HSA-QTP system was studied using spectroscopic and molecular docking techniques. The UV-Vis absorption study shows hyperchromicity in the spectra of HSA on the addition of QTP, suggesting the complex formation and interactions between QTP and HSA. The results of intrinsic fluorescence indicate that QTP quenched the fluorescence of HSA and confirmed the complex formation between HSA and QTP, and this quenching mechanism was a static one. Thermodynamic analysis of the HSA-QTP system confirms the involvement of hydrophobic forces, and this complex formation is spontaneous. The competitive displacement and molecular docking experiments demonstrated that QTP is preferentially bound to HSA subdomain IB. Furthermore, the CD experiment results showed conformational changes in the HSA-QTP system. Besides this, the addition of QTP does not affect the esterase-like activity of HSA. This study will help further understand the credible mechanism of transport and delivery of QTP via HSA and design new QTP-based derivatives with greater efficacy.

## 1. Introduction

In recent years, psychoactive drug usage has increased worldwide due to the increasing incidence of related psychiatric disorders [1]. However, the most commonly prescribed psychoactive drugs, such as antidepressants, antipsychotics, and mood stabilizers, cause unwanted side effects (excessive systemic drug exposure) and toxicity to human systems [2,3]. 

**Figure 1:**
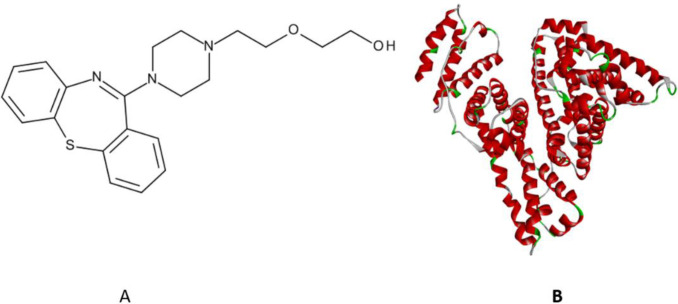
(**A**) chemical structure of QTP; (**B**) molecular structure of HSA.

Quetiapine (QTP Figure 1A) is a second generation (short-acting atypical) antipsychotic drug of dibenzothiazepine (class), which is used to treat schizophrenia, acute bipolar disorder, and major depression in adolescents and adults [4,5,6,7]. The exact mechanism of action of QTP is poorly understood. However, QTP is an antagonist of various neurotransmitter receptors in the brain, such as dopamine D_1_ and D_2_, adrenergic alpha receptors alpha_1_ and alpha_2_, histamine H_1_, and serotonin 5-HT_1A_ and 5-HT_2_, respectively [8,9]. Specifically, the antipsychotic and antidepressant effects of QTP are believed to be due to the interactions of the above-mentioned neurotransmitter receptors dopamine (D_1_ and D_2_), adrenergic alpha receptors (α_1_ and α_2_), histamine (H_1_), and serotonin (5-HT_1A_ and 5-HT_2_) [9]. 

Recent studies explore the insight of binding affinity and mechanism of plasma proteins and drug interactions [10,11,12,13,14,15]. Recently, nanotechnology has helped explore the interaction mechanisms [16,17]. However, the interaction between the drug proteins (plasma) and their mechanism is vital because they directly affect therapeutic drugs’ pharmacodynamic and pharmacokinetic properties in the human system [10]. Moreover, the drug proteins (plasma) interactions help to decipher the therapeutic efficacy, distribution, and bioavailability of therapeutic drugs and assist in enhancing solubility in plasma protein, reducing toxicity, and protecting against oxidation [18,19,20]. 

Human serum albumin (HSA) is a principal plasma protein with critical physiological functions and facilitates the transportation of many molecules and metabolites (Figure 1B) [21]. It is a monomeric chain globular plasma protein (585 amino acids residues), and its 3D structure consists of three homologous domains (I-III-A and B subdomains). The essential binding regions for drugs in the HSA are Sudlow’s site I (subdomains IIA) and Sudlow’s site II (subdomains IIIA) [22,23,24,25]. However, there is also Site III (subdomain IB), which is also believed to play an essential role in binding various drugs [26]. Therefore, HSA has multiple binding sites and can bind several different drugs, thus making it a fundamental functional drug carrier [27]. Furthermore, the binding of therapeutic drugs within HSA is commonly reversible via weak interactions such as hydrogen bonding, hydrophobic forces, ionic interactions, and van der Waal’s interactions [28].

To the best of our knowledge, the interaction binding mechanism of QTP and HSA has still not been investigated. Here, multi-spectroscopic techniques and biochemical and molecular docking approaches were applied to scrutinize the binding properties of QTP with HSA under physiological conditions. However, we considered the possibility of complexation between QTP-HSA, which would explore the pharmacodynamics and pharmacokinetics of QTP. The QTP-HSA interactions reported here would explain the binding mechanism at the molecular level and facilitate efforts to modify new therapeutic drugs that optimize their distribution within the human body.

## 2. Results and Discussion

### 2.1. UV-Vis Absorption Spectroscopy

**Figure 2:**
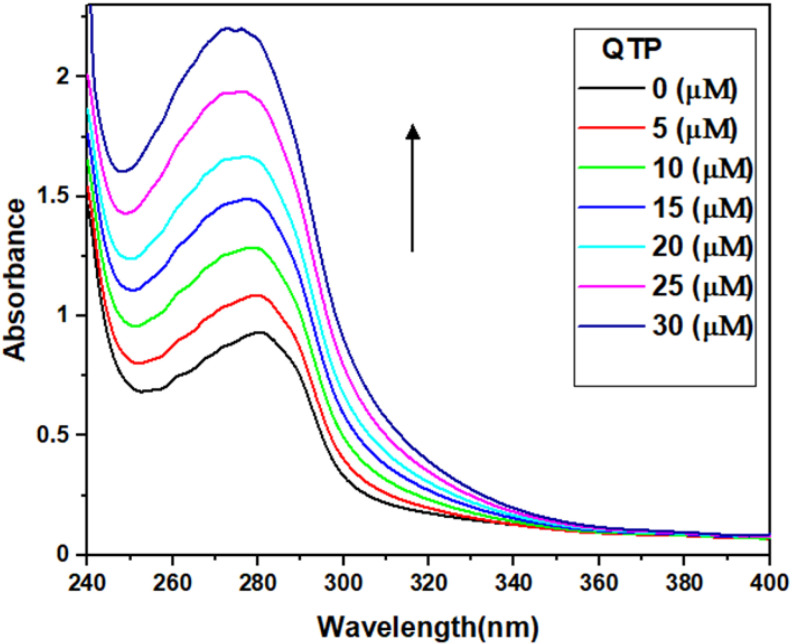
UV absorption spectra of HSA (5 μM) in the absence and presence of increasing concentrations of QTP (5–30 μM) in the wavelength range 240–410 nm.

UV-Vis spectral analyses are carried out to observe the structural and conformational changes in the protein molecule induced by the binding ligands and thus to obtain information about their interaction mechanism. [29]. The UV-Vis absorption spectra of the HSA and HSA-QTP complex are shown in Figure 2. It is apparent from the spectra that HSA exhibits an absorption peak at 280 nm coming from the π-π* transition of the aromatic amino acids (tryptophan (W), tyrosine (Y), phenylalanine) [30]. An increase in QTP concentration was accompanied by a slight shift in the absorption wavelength. This blue shift indicates that QTP binding is associated with changes in the local environment of HSA. In addition, there is an increase in UV-absorption intensities of HSA at around 280 nm at increasing concentrations of QTP, and this hyperchromicity suggests the HSA-QTP system formation. Hyperchromicity at around 280 nm in HSA after QTP addition also confirms that the aromatic amino acid (W and Y) microenvironment changes due to the HSA-QTP complex formation [31,32]. 

### 2.2. Fluorescence Emission Spectroscopy of the HSA-QTP Complex 

**Figure 3:**
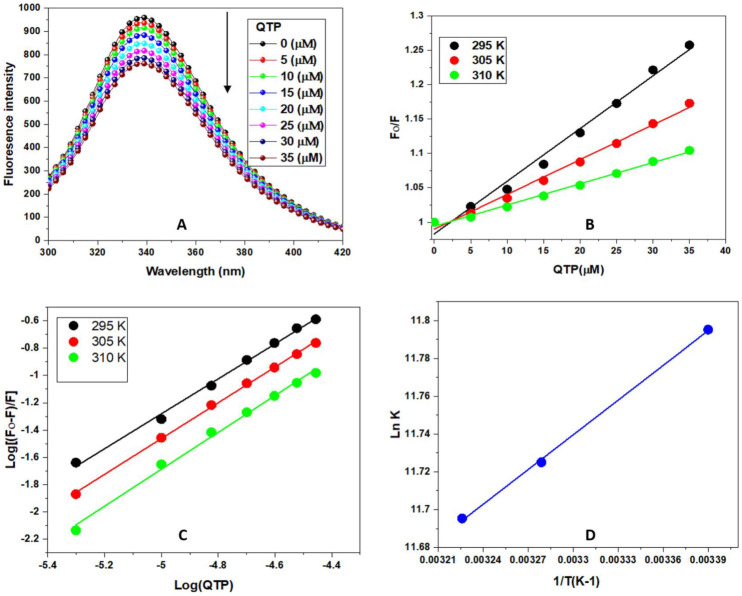
(**A**) Steady-state fluorescence emission spectra of HSA were recorded in the absence and presence of increasing concentrations of QTP. The intrinsic fluorescence of the HSA was measured at 295 K in the wavelength range of 300–420 nm after exciting at 295 nm. The black arrow represents fluorescence quenching of HSA on titration with QTP (**B**) Stern-Volmer plot for QTP-HSA interaction (295, 300, 310 K). (**C**) Double log plot for the QTP-HSA interaction at different temperatures (295, 300, 310 K). (**D**) van ’t Hoff plot (lnK vs. 1/T) for the binding of QTP to HSA. The concentration of HSA was 5 μM and was titrated with QTP (0–35 μM) in all the experiments (**A**–**D**).

Fluorescence emission spectroscopy is a multipurpose biophysical technique used to study the binding mechanism of protein-ligand interactions and to evaluate the binding parameters [10,12,26]. The fluorescence emission spectra of HSA alone and the HSA-QTP complex are given in Figure 3A. It is apparent from Figure 3A that HSA exhibits a strong emission peak at 340 nm upon excitation at 295 nm due to W-214 residue. Further, the addition of different concentrations of QTP (0–35 μM) leads to the quenching of HSA fluorescence intensity without changing the peak shape. This fluorescence quenching suggests the formation of the HSA-QTP system and suggests a possible microenvironmental alteration in HSA upon treatment with QTP [33,34].

#### 2.2.1. Fluorescence Quenching Mechanism (FQM) of the Interactions of the HSA-QTP System

According to the literature, the protein’s fluorescence quenching mechanism (FQM) consists mainly of dynamic quenching and static quenching. In the case of dynamic quenching, the interaction of the fluorophore with the quencher is indirect. In contrast, in the case of static quenching, a ground state complex formation exists between the fluorophore and quencher [30]. Therefore, the FQM can be sorted out based on their temperature dependence. Furthermore, in the case of static quenching, K_sv_ values are inversely proportional to temperature, whereas in dynamic K_sv_, the values are directly proportional to temperature. Therefore, the FQM of the HSA-QTP system was evaluated by recording the fluorescence spectra of HSA-QTP at different temperatures (295, 300, and 305 K), and the fluorescence quenching data of the HSA-QTP system was analyzed using the Stern-Volmer equation [30]:

(1) $$\frac{F_{0}}{F}=1+K_{\mathrm{sv}}\left[Q\right]$$

where F_0_ and F represent the steady-state fluorescence of HSA and the HSA-QTP complex, respectively. [Q] represents the quencher concentration (QTP), and K_sv_ represents the Stern-Volmer constant. The K_sv_ plot for the HSA-QTP system obtained at various temperatures (295, 300, and 305 K) is given in Figure 3B. It is found that the K_sv_ values for the HSA-QTP system decreased with a temperature rise, confirming the static quenching mechanism for the HSA-QTP system (Table 1). In addition, the fluorescence mechanism (quenching) was also analyzed according to the bimolecular rate constant values using the equation:

(2) $$k_{q}=K_{\mathrm{sv}}/\tau_{0}$$

where k_q_ is the bimolecular rate constant, and τ_0_ is the average lifetime of the protein in the absence of the quencher and is valued at 10^−8^ for biopolymers [35]. The calculated bimolecular quenching rate constant value for the HSA-QTP system is presented in Table 1. The k_q_ values were found to be higher than the value of the scattering collision constant (2 × 10^10^ M^−1^ s^−1^), which again suggests the involvement of a static quenching mechanism between the HSA-QTP system [36].

#### 2.2.2. Evaluation of the Binding Constants and the Number of Binding Sites in the HSA-QTP System 

Intrinsic fluorescence data at different temperatures (295, 300, and 305 K) were used to determine the binding constant (K_b_) and binding stoichiometry (n) of the HSA-QTP system by using the following equation [10,30]:

(3) $$\log\frac{\left(F_{0}-F\right)}{F}={\log\;K}_{b}+n\;\log\left[Q\right]$$

where F_0_ and F represent fluorescence intensities of HSA with or without the quencher (QTP), respectively. K_b_ and n represent the binding constant and binding stoichiometry in the HSA-QTP system. The double log plot of log [(F_0_ − F)/F] vs. log [Q] (Figure 3C) was used for the determination of the binding constant and binding stoichiometry. The values of K_b_ and n were calculated from the intercept and slope of the plot, as shown in Figure 3C. As per Figure 3C, K_b_ and n at different temperatures for the HSA-QTP system are presented in Table 2. A decrease in the binding constant was observed at higher temperatures for the HSA-QTP system. Further, the binding constants were ~10^4^, suggesting a moderate binding between HSA and QTP. 

#### 2.2.3. Determination of the Binding Forces between HSA and QTP-Thermodynamic Analysis 

The primary binding intermolecular forces that are involved in the drug-protein interactions were estimated via thermodynamic parameters. The protein-drug interactions are held together by hydrophobic interactions, hydrogen bonds, electrostatic forces, and van der Waal interactions. Moreover, the sign and magnitude of the enthalpy (ΔH^0^) and entropy (ΔS^0^) change to determine the nature of binding forces in the drug-protein complex. For the hydrophobic interactions, the sign and magnitude must have a positive value for ΔH^0^ and ΔS^0^. At the same time, in the case of van der Waals forces and hydrogen bonding, it must be negative for ΔH^0^ and ΔS^0^ [36,37]. Additionally, for the electrostatic interaction, ΔH^0^ should be negative and ΔS^0^ positive. The free energy (ΔG^0^) change of the HSA-QTP system can be determined by using the van ’t Hoff equation and the thermodynamic equation given below:

(4) $${\mathrm{lnK}}_{b}=-\frac{\Delta H^{0}}{\mathrm{RT}}+\frac{\Delta S^{0}\;}{R}$$

(5) $$\Delta G^{0}=\Delta H^{0}-T\Delta S^{0}$$

where R represents the gas constant (8.314 J mol ^−1^ K ^−1^), T is the temperature in kelvins, and K_b_ represents the binding constant at the studied different temperatures. ΔH^0^ and ΔS^0^ are obtained from the slope and intercept of the plot between lnK and 1/T (Figure 3D). The results of ΔG^0^, ΔH^0^, and ΔS^0^ obtained from HSA-QTP interactions are summarized in Table 3. The positive values of ΔH^0^ and ΔS^0^ for the HSA-QTP system suggest that hydrophobic interactions played a significant role in the binding process of QTP to HSA. Thus, the formation of the HSA-QTP complex was exothermic and spontaneous [38]. 

#### 2.2.4. Synchronous Fluorescence Spectroscopy (SFS) Experiment

**Figure 4:**
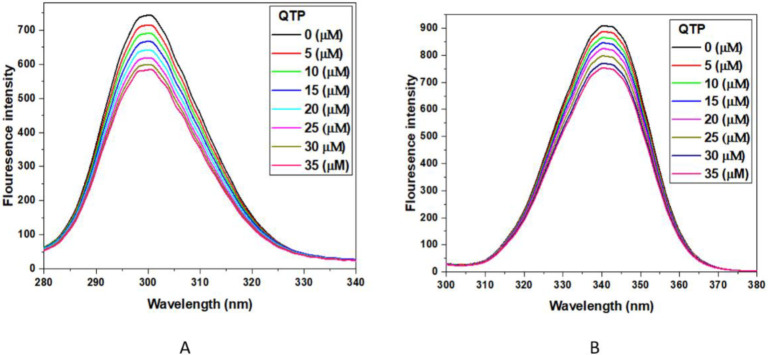
Synchronous fluorescence spectra at Δλ = 15 nm (**A**) and Δλ = 60 nm (**B**) of HSA (5 μM) in the absence and presence of increasing concentrations of QTP (0-35 μM). At Δλ = 15 nm (for Y), the excitation wavelength of HSA was fixed at 240 nm, and the emission range was 255–400 nm, whereas at Δλ = 60 nm (for W), the excitation wavelength was taken at 240 nm and the emission range was 300–400 nm.

The synchronous fluorescence spectrometry helps to provide information about the local environment of proteins around W and Y residues upon interaction with ligands [30,39]. In this experiment, the fluorescence difference between excitation and emission wavelengths reflects the nature of the spectra. A difference of wavelength (Δλ) of 15 nm is characteristic for (Y), and 60 nm is typical of (W) residues. Therefore, any shift in the maximum emission wavelength reflects the local environment changes around aromatic amino acid residue (Y and W) [40]. The SFS emission spectra of the HSA-QTP complex are given in Figure 4A,B. It was clear from Figure 4 that the HSA fluorescence intensity of both (W and Y) regularly decreases with the addition of QTP. Further, no shift in the emission wavelength was observed for either of the spectra at Δλ = 15 nm or 60 nm. The HSA-QTP interaction did not lead to any microenvironmental change in the protein molecule upon interaction.

#### 2.2.5. Binding and Prediction of Site Markers in the HSA-QTP System

A site marker displacement experiment was investigated to identify QTP binding site on HSA. In this experiment, here warfarin (WAR) for Sudlow’s site I (subdomain IIA), ibuprofen (IBU) for Sudlow’s Site II (subdomain IIIA), and hemin (HEM) for binding site III (subdomain IB) were used as HSA site marker probes; [10,26]. As a result, fluorescence spectra were recorded HSA-QTP system in the presence of site marker probes (0–30 μM). Moreover, the displacement percentage (I%) of QTP with the site markers is estimated by the following methods [40,41]:

(6) $$I\left(\%\right)=\frac{F_{2}}{F_{1}}\times 100$$

**Figure 5:**
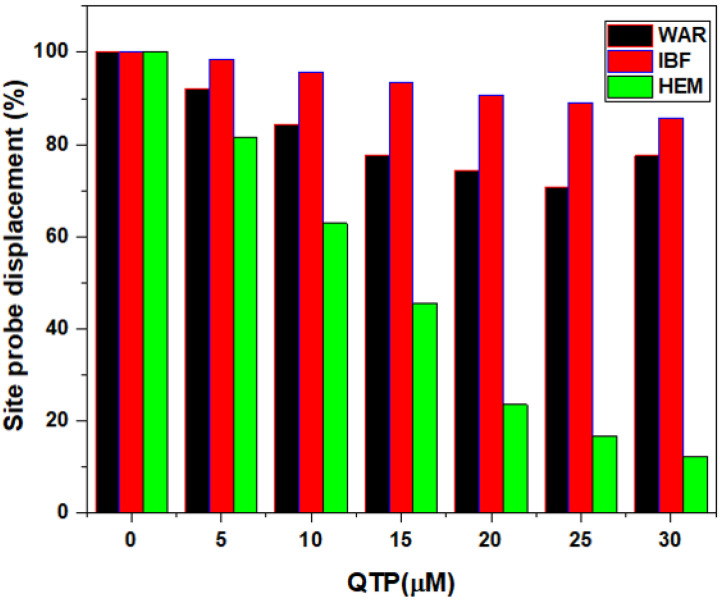
Effect of site probes on the fluorescence emission intensities of the HSA-QTP system. The experiments were carried out using three site probes (warfarin, ibuprofen, and hemin). (HSA = 5 µM, QTP = 10 µM, C = 0-30 µM), λex = 295 nm, T = 295 K.

F_1_ and F_2_ represent the fluorescence emission intensities of the HSA-QTP system in the absence and presence of different site markers, respectively. However, the percentage of displacement values of the HSA-QTP complex against the different concentrations of site markers is shown in Figure 5. It is apparent from Figure 5 that the displacement percentage of QTP from HSA by hemin is appreciably higher than WAR and IBU. Thus, the binding site of QTP is predicted to be in site III (subdomain IB) of HSA.

#### 2.2.6. Circular Dichroism Spectra Changes in HSA upon QTP Binding 

**Figure 6:**
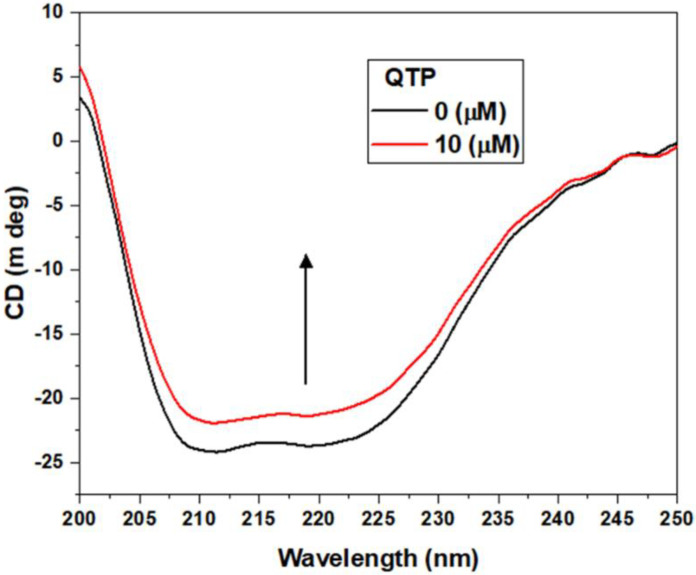
Circular dichroism spectra of HSA (5 μM) in the absence and presence of QTP (10 μM).

Circular dichroism (CD) spectroscopy is a versatile technique mainly used to detect structural and conformational changes in protein structure. The CD spectra of HSA have two negative peaks in the UV region, which reflect α-helix at around 208 and 222 nm of the protein [42]. Figure 6 represents the CD spectra of HSA alone and the HSA-QTP system at different molar ratios of 1:0–1:2. The addition of QTP leads to a decrease in the ellipticity of HSA, suggesting the loss of α-helical content. The CD results showed that the α-helix content of the HSA and QTP-HSA system was 55.92% and 48.88%, respectively. Therefore, these results suggest that the addition of QTP leads to secondary structure change of HSA α-helix content. 

#### 2.2.7. QTP-Induced Thermal Stabilization of HSA

**Figure 7:**
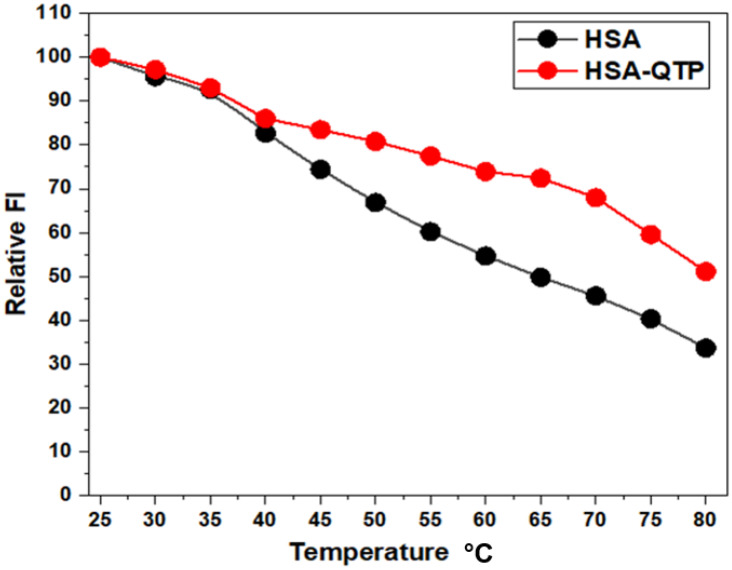
Thermal stability profiles of HSA and the QTP-HSA (1:10) system in the temperature range, 25–80 °C, as monitored by fluorescence intensity measurements at 343 nm (FI 343 nm) using a protein concentration of 5 μM in 60 mM sodium phosphate buffer, pH 7.4.

The binding of drugs to plasma proteins can increase the protein’s thermal stability [43]. Various studies have shown that drugs induced thermal stabilization to HSA [44,45]. Therefore, the thermal stability measurements of HSA were carried out at different temperatures in the absence and presence of QTP binding. The temperature-dependent titrations measurements were performed on HSA (5 µM) without or with QTP (50 µM) in different temperature range, 25–80 °C (5 °C intervals). Figure 7 shows the influence of temperature on the fluorescence intensity of the HSA and HSA-QTP system at 343 nm. In the presence of QTP at 45 °C, the decrease in FI of the HSA-QTP system was lesser than HSA alone. However, our thermal stability results demonstrated QTP-induced stability to HSA via QTP-HSA system formation (coupling of binding and unfolding equilibrium) [46]. 

#### 2.2.8. Effect of QTP Binding on the Esterase-Like Activity of HSA

**Figure 8:**
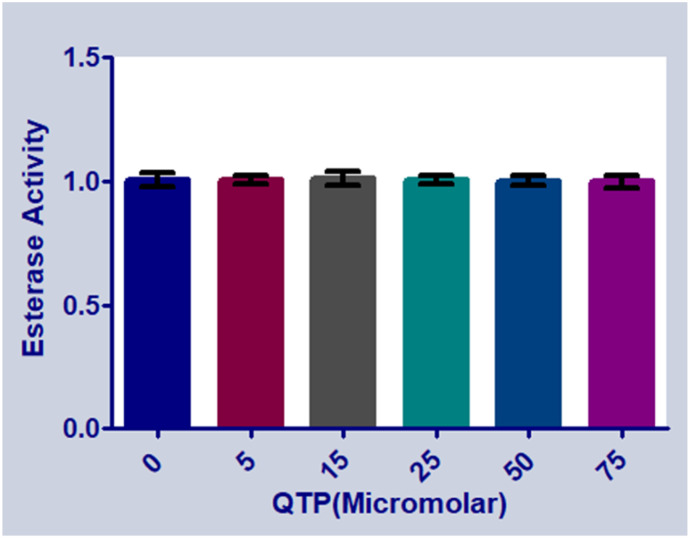
Estimated esterase activity in HSA (5 μM) in the absence and presence of increasing concentrations of QTP (0–75 μM).

HSA is the most abundant protein in the blood plasma and possesses catalytic functions such as esterase-like activity [47]. Amino acid residues such as Arg-410 Tyr-41 (Sudlow’s site II (subdomain IIIA)) of HSA play a predominant function in esterase activity (Watanabe et al., 2000) [48]. However, the effect of QTP binding on the esterase-like activity of HSA is shown in Figure 8. It was observed that upon the addition of QTP (0–75 μM), there is no inhibiting effect on the esterase-like activity of HSA. 

#### 2.2.9. Computational Modeling of the HSA-QTP Complex

**Figure 9:**
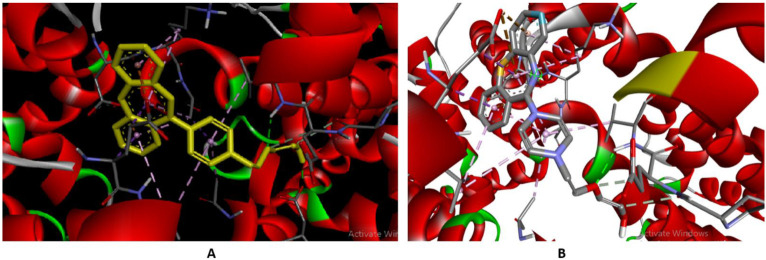
(**A**) Molecular models of HSA complex with QTP. (**B**) Detailed view of the docking poses of the HSA-QTP complex. Selected protein side-chains are shown as ribbons.

**Figure 10:**
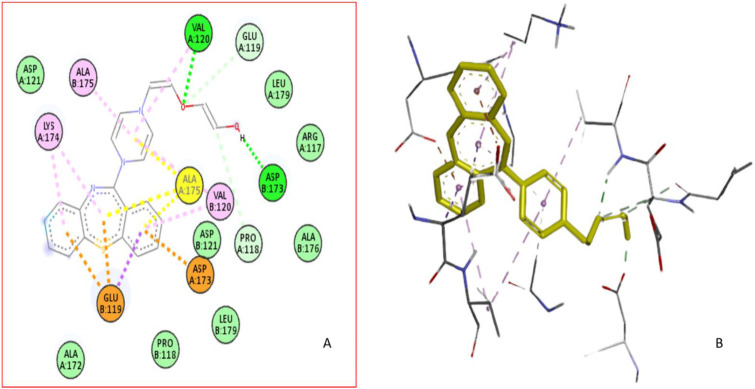
(**A**) The 2D binding site was magnified to show the surrounding amino acid residue of HSA interacting with QTP. (**B**) Three-dimensional structure of interactions of HSA with QTP.

The binding region and amino acid residues involved in the interaction of QTP with HSA were evaluated by molecular docking analysis [27,45,47]. The most suitable confirmation of the HSA-QTP system is given in Figure 9A,B. The molecular docking results suggested the QTP binding region at subdomain IB (Site III) of HSA (Figure 9A). Further, QTP binds to HSA and forms two hydrogen bonds with VAL120 and ASP173 amino acid residues of HSA (Figure 10A). In addition to the two hydrogen bonds, the QTP molecule is surrounded by LEU-179, ARG-117, ALA-176, ASP-121, LEU-179, PRO-118, ALA-172, VAL-120, ASP-173, GLU-119, LYS-174, and ALA-175 through different interactions (Figure 10). The autodock results also showed that the binding affinity of QTP to HSA was −8.2 kcal mol^−1^. Thus, we can conclude that the molecular docking results agree with the site displacement markers experiments (Figure 5). 

## 3. Materials and Methods 

### 3.1. Chemical Reagents

HSA (A1887, fatty acid and globulin free) and QTP (purity, 90%) were obtained from Sigma Chemical Co. (St. Louis, Mo, USA) and GLR. Scientific. Co. (Delhi, India), warfarin, ibuprofen through the National Scientific company (Riyadh, KSA) and hemin were obtained from SRL Pvt. Ltd. (Mumbai, India). All other chemicals and reagents for this study were of high analytical grade.

### 3.2. Sample Preparation

HSA stock solution (200 µM) was prepared in Tris-HCI buffers (0.2 M) pH 7.4. In addition, the stock of QTP (10 mM) was prepared in methanol and then diluted with Tris-HCI buffers (0.2 M), pH 7.4, to prepare the working standard samples of QTP. Finally, the buffer was prepared using Type I Millipore water (Burlington, MA, USA).

### 3.3. Instrumentations

The UV-Vis absorption spectra were recorded on a UV-1800 spectrophotometer (Shimadzu, Kyoto, Japan) using a 1.0 × 1.0 cm cell. The fluorescence experiments were recorded on an RF-5301PC spectrofluorometer (Shimadzu, Kyoto, Japan) fitted with a xenon-flash lamp with quartz-cuvettes. The circular dichroism experiments were recorded on a JASCO J-1500-CD spectrophotometer (Mary’s Court, Easton, MD, USA) equipped with a Peltier temperature controller with a quartz cuvette. 

### 3.4. Methods

#### 3.4.1. UV-Visible Absorption Spectroscopy

UV-Visible absorbance spectra of HSA (5 μM) in the absence and presence of QTP (0–30 μM) at 298 k were recorded at wavelengths from 240 to 410 nm, and baseline correction was performed using an appropriate buffer. 

#### 3.4.2. Steady-State Fluorescence Measurements 

The intrinsic fluorescence spectra of HSA were recorded at an emission wavelength (300–420 nm) upon excitement at 295 nm. The HSA (5 μM) samples were titrated with QTP (0–35 μM) at three different temperatures (295, 300, 305 K) to estimate thermodynamic parameters. The obtained fluorescence data were corrected for inner filter effects.

#### 3.4.3. Synchronous Fluorescence Spectroscopy (SFS) Experiments

For this experiment, SFS measurements of HSA (5 μM) titrated with different concentrations of QTP (0–35 μM) were performed in different experiments by setting wavelength intervals (Δλ) at 15 nm for tyrosine residue (Y) and 60 nm for tryptophan residue (W) in the same experimental conditions as the fluorescence measurements.

#### 3.4.4. Competitive Site Probe Displacement (CSPD) Experiments

Briefly, in these experiments, CSPD experiments were carried out to locate the binding site of QTP on the HSA. Warfarin (WAR) (Sudlow’s site I), ibuprofen (IBU) (Sudlow’s site II), and hemin (HEM) (site III) site markers were used to locate the binding region of QTP in HSA. Initially, fluorescence spectra were performed by titrating a solution of 5 μM HSA and QTP 10 µΜ with increasing site marker (0–30 µΜ) concentrations in separate experiments. All other parameters (excitation and emission wavelength) were uniform for the fluorescence measurements.

#### 3.4.5. Circular Dichroism (CD) Spectroscopy Measurements

The far CD spectra of HSA and the HSA-QTP complex were recorded at a wavelength between 200 and 260 nm on the spectropolarimeter with a scan speed of 100 nm min^−1^. HSA (5 μM) was titrated with 10 μM QTP. The measured ellipticity values were expressed as the mean residue ellipticity (MRE) in deg cm^2^ dmol^−1^, defined by equation [48]:

(7) $$\mathrm{MRE}=\frac{\mathrm{Observed}\;\mathrm{CD}\;\left(\theta \mathrm{obs}\right)\;}{c\times n\times l\times 10}$$

where θobs is the measured ellipticity in millidegree, “n” is the number of amino acids residues, “l” is the path length of the cuvette (cm), and “c” is the molar concentration of protein. The α-helical content of HSA was determined by equation [48]:

(8) $$\;\alpha \text{-}\mathrm{helical}\;\mathrm{content}\left(\%\right)=\frac{\left[{\mathrm{MRE}}_{208}-4000\right]}{\left[33,000-4000\right]}\times 100$$

where MRE_208_ is the mean residue elasticity (MRE) at 208 nm.

#### 3.4.6. Thermal Stability Studies of HSA and the HSA-QTP System

The thermal stability of HSA without and with QTP was investigated using fluorescence measurements. The fluorescence spectra of the HSA (5 µM) and HSA-QTP (50 µM) complex were recorded (300–400 nm upon excitation at 295 nm) in the temperature range 25–80 °C (with 5 °C intervals). The solution mixture (HSA-QTP) was incubated for 1 h at 25 °C before fluorescence measurements. 

#### 3.4.7. HSA Esterase Activity (E.A.) Assay 

The influence of QTP on the esterase activity of HSA was investigated by estimating the formation of p-nitrophenol [40]. The E.A. analysis is based on the fact that 4-nitrophenyl acetate (P-NPA) interacts with HSA and generates 4-nitrophenol (maximum absorption at 400 nm) [49,50]. For this experiment, the concentration of P-NPA (5 μM) and HSA (5 μM) was fixed, and the concentration of QTP increased (0–75 μM).

#### 3.4.8. Molecular Docking between HSA and QTP

The mechanism of QTP binding with HSA has been predicted by molecular docking using AutoDock Vina [51]. The molecular structure of HSA (PDB ID: 1AO6) and QTP (Chem-Spider ID 4827) was obtained from Protein Data Bank (PDB) and Chem-spider, respectively. In the docking protocol, a grid box size of 60 × 60 × 60 with coordinates set to x = 45, y = 12, and z = 18 was built to cover the entire protein. All other parameters were maintained to the default setting. The docked structure of the HSA-QTP system was analyzed with Discovery studio.

## 4. Conclusions

In the present study, the antipsychotic drug QTP was characterized for its binding interaction to HSA using spectroscopic and biochemical methods and computational approaches. The results obtained from the QTP-HSA binding interactions showed moderate binding affinity of QTP toward HSA. In addition, the involvement of hydrogen bonding and hydrophobic interactions was observed. The spectroscopic studies suggest a complex formation between QTP and HSA, and the system follows a static quenching mechanism. Conversely, the thermodynamic parameters of the HSA-QTP system calculated via fluorescence spectroscopy at different temperatures indicate a spontaneous and exothermic process and indicate the predominant forces to be hydrophobic interactions.

Further, the site-displacement assay and molecular docking results confirm the QTP binding region at subdomain IB of HSA. The CD spectra and UV-Vis spectroscopy identified changes in the secondary structure of HSA upon its interaction with QTP. In addition, QTP did not inhibit the esterase-like activity of HSA. This study is essential and is expected to help understand the drug’s mechanisms and pharmacokinetics for further clinical research and novel drug delivery systems.

## Figures and Tables

**Table 1:** The values of the Stern-Volmer constant and quenching rate constant for the QTP-HSA system.

pH	Temp (K)	K_sv_ (× 10^4^ M^−1^)	K_q_ (× 10^12^ M^−1^ s^−1^)	R²
7.4	295	0.7	0.7	0.987
	305	0.5	0.5	0.992
	310	0.3	0.3	0.993

**Table 2:** The binding constant values and the number of binding sites for the interaction of QTP with HSA.

pH	Temp (K)	K_b_ (× 10^4^ M^−1^)	N	R²
7.4	295	1.326	1.28	0.996
	305	1.236	1.31	0.994
	310	1.200	1.35	0.994

**Table 3:** Various thermodynamic parameters for QTP-HSA complex formation at various temperatures.

Temp (K)	ΔH^0^ (KJ mol^−1^)	ΔS^0^ (JK^−1^ mol^−1^)	TΔS^0^ (KJ mol^−1^)	ΔG^0^ (KJ mol^−1^)
295	5.087	81	23.89	−18.8
305			24.705	−19.61
310			25.11	−20.11

## Data Availability

Data will be available on request to corresponding author.
